# Interstage Pressures of a Multistage Compressor with Intercooling

**DOI:** 10.3390/e23030351

**Published:** 2021-03-15

**Authors:** Helen Lugo-Méndez, Teresa Lopez-Arenas, Alejandro Torres-Aldaco, Edgar Vicente Torres-González, Mauricio Sales-Cruz, Raúl Lugo-Leyte

**Affiliations:** 1 Departamento de Procesos y Tecnología, Universidad Autónoma Metropolitana—Cuajimalpa, Av. Vasco de Quiroga 4871, Santa Fé, Cuajimalpa, Ciudad de México 05348, Mexico; hlugo@cua.uam.mx (H.L.-M.); mtlopez@cua.uam.mx (T.L.-A.); asales@cua.uam.mx (M.S.-C.); 2 Departamento de Ingeniería de Procesos e Hidráulica, Universidad Autónoma Metropolitana—Iztapalapa, Av. San Rafael Atlixco 186, Vicentina, Iztapalapa, Ciudad de México 09340, Mexico; ata@xanum.uam.mx (A.T.-A.); etorres@xanum.uam.mx (E.V.T.-G.)

**Keywords:** interstage pressures, multistage compressors, intercooling

## Abstract

This paper considers the criterion of minimum compression work to derive an expression for the interstage pressure of a multistage compressor with intercooling that includes the gas properties, pressure drops in the intercoolers, different suction gas temperatures, and isentropic efficiencies in each compression stage. The analytical expression for the interstage pressures is applied to estimate the number of compression stages and to evaluate its applicability in order to estimate interstage pressures in the operation of multistage compressors, which can be especially useful when their measurements are not available.

## 1. Introduction

Gas compression is widely used in many fields, from internal combustion engines and industrial processes to domestic gas supply and refrigeration [1]. High pressures are required to overcome pressure drops due to friction in gas pipelines, to reach an equilibrium condition for separation processes, to increase a specific reaction rate, to improve the yield of a chemical reaction, or to avoid parallel reactions. Low temperatures for the cold treatment of metals, refrigeration, air-conditioning, or the liquefaction and separation of gases, make the multistage vapor compression systems important to study [2,3,4].

Minimum work occurs for an ideal isothermal compression; however, this process requires an infinite number of intercoolers. In real conditions, for design purposes and to approach the lowest energy consumption, the compression ratio is split in two or more stages, cooling the compressed gas in between [5]. For a compression process, the criterion of minimum work is one of the most commonly used criteria to determine the optimal sequence of interstage pressures and, therefore, the location and optimal number of intercoolers. In fact, this is only a partial criterion of optimization for the optimal number of intercoolers, which can, however, be used as an upper limit. In practice, the final decision to establish the number of intercoolers depends primarily on the overall pressure ratio and the compressor capacity and should be determined by incorporating techno-economical criteria [6,7].

In classical thermodynamics, the interstage pressure of an ideal gas minimizing the compression work of a two-stage compressor with intercooling corresponds to the geometric mean of the suction and discharge pressures [8,9,10,11]. This well-known relation assumes that the compressors operate isentropically and discard the intercooler pressure drops, and that the temperature of the compressed gas at the beginning of each compression stage is the same. With these same assumptions, Hernández et al. [12] report an expression for the interstage pressures for a compressor of more than two stages to evaluate the performance of a regenerative gas turbine cycle. It is important to point out that when ideal conditions are assumed, the optimum interstage pressure does not depend on the gas properties. Vadasz and Weiner [6] find the optimal interstage pressures, which are dependent on gas properties, for a general compression process with intercooler pressure drops and a temperature difference between the compressed gas at the intercooler outlet and the global suction state. These authors also establish a numerical approach to specify the optimal location and number of intercoolers. Recently, López-Paniagua et al. [13] employed Lagrange multipliers to determine the optimum interstage pressures for a multistage compression process with different-stage isentropic efficiencies. Their result is applied to the design of a multistage compression plant with reciprocating compressors.

The use of the geometric mean of the suction and discharge pressures has been mainly applied in performance analysis, design, and optimization of refrigeration systems with dual stage compressors [14,15,16] and more recently in the analysis and optimization of two-stage transcritical carbon dioxide cycles for heating applications [17]. Manole [18] shows that for a CO_2_ refrigeration cycle, the estimated interstage pressure from the suction and discharge pressures geometric mean underestimates the actual interstage pressure of the cycle. Jekel and Reindl [10] explore single- versus two-stage compression arrangements from an operating efficiency perspective. They find that the optimum operating efficiency for each system is obtained when the real interstage pressure is smaller than that obtained from the geometric mean. Özgür [11] and Romeo et al. [19] use directly and indirectly the geometric mean as the basis for their initial designs used in their performance studies of refrigeration cycles with two and three compression stages with intercooling, respectively. Srinivasan [20] shows that the criterion of equal discharge temperatures of each stage is a good criterion for the choice of interstage pressure for CO_2_ compressors used in low (−30 °C) and medium temperature (−5 °C) refrigeration. Lugo-Leyte et al. [21] study the performance of complex gas turbine cycles with multistage compression. They determined that the optimum pressure ratios are in an acceptable range, between 8.1 and 23.1 for the maximum power and between 17.4 and 32.2 for the maximum thermal efficiency. Lewins [22] models and optimizes a two-stage compressor with an intercooler considering the ideal gas model. He uses the Lagrange optimization method to find the operating conditions to achieve the maximum work in the gas turbine. Furthermore, he shows the optimum condition can be calculated based on the isentropic efficiencies of the compressors and the efficiency of the intercoolers. Azizifar and Banooni [23] model and optimize the power consumption of a two-stage compressed air system considering the ideal gas model. The system includes two centrifugal compressors, a casing, and a tube intercooler. The power consumption is expressed in terms of the isentropic efficiencies and thermal effectiveness of the intercooler. The isentropic efficiencies of the compressors are considered as functions of the inlet temperature, and the thermal effectiveness of the intercooler is considered as a function of the inlet air temperature, inlet water temperature of the intercooler, and inlet volumetric air flow rate of the system.

This paper considers the criterion of minimum compression work to determine an analytical expression for the interstage pressures of a multistage compressor with intercooling, taking into account the properties of the gas, pressure drops in the intercoolers, different suction gas temperatures, and isentropic efficiencies in each compression stage. The derivation of the expression is pursued in two ways: in Section 3, by identifying that the product of the interstage pressure ratios and the coefficient ${\left(T_{2j-1}/\eta_{SIC,j}\right)}_{j}^{(\gamma -1)/\gamma }$ remain constant for all the compression stages; in Appendix B, by carrying out successive substitutions to solve the system of recursive nonlinear equations that define the interstage pressure ratios, followed by a mathematical induction proof presented in Appendix D to prove the expression for the optimal interstage pressures for any number of compression stages. The obtained expression is applied in Section 4 to estimate the number of compression stages of a multistage compressor, showing the usefulness of the expression in compressor design and sizing, and to estimate the interstage pressures of an off-design two-stage centrifugal compressor handling natural gas with intercooling and phase separators, revealing that the expression can be valuable in monitoring and diagnosis of such systems, especially when properties of gas and measurements of interstage pressures are not available.

## 2. System Description and Assumptions

**Figure 1:**
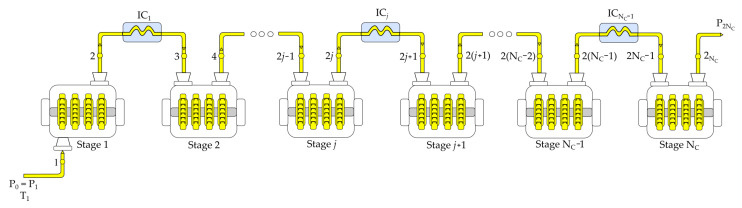
Schematic diagram of an $N_{c}$-multistage compressor with intercooling.

**Figure 2:**
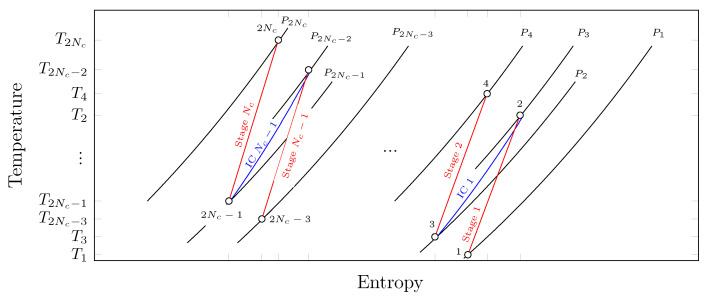
Temperature–entropy diagram of an $N_{c}$-multistage compression process with intercooling.

We consider the $N_{c}$-multistage compression system presented in Figure 1 that is composed of $N_{c}$ compressors alternated with $N_{c}-1$ intercoolers. In this system, the gas is compressed from the suction state $\left(T_{s}=T_{1},P_{s}=P_{0}=P_{1}\right)$ to the discharge state $\left(T_{d}=T_{2N_{c}},P_{d}=P_{2N_{c}}\right)$. In each *j*-compression stage, the fluid is compressed from the state 2j−1 to the state 2j, and then the fluid goes through a cooling process from the state 2j to the state 2j+1, as shown in Figure 2.

The main characteristics of the $N_{c}$-multistage compression system considered in this work are as follows:

- A constant mass flow rate of a working fluid behaving as an ideal gas with constant heat capacities is compressed.
- The gas undergoes a pressure drop in each *j*-intercooler—see Figure 2. The pressure drop coefficient across the *j*-intercooler is defined as
  (1) $$\varepsilon_{j}=\frac{P_{2j}-P_{2j+1}}{P_{2j}},\;\;\mathrm{for}\;\;j=1,\ldots ,N_{c}-1$$
  where $P_{2j}$ and $P_{2j+1}=P_{2\left(j+1\right)-1}$ denote the inlet and outlet pressures for the *j* intercooling process, and they also correspond to the discharge pressure of the *j* compressor and the suction pressure of the j+1 compressor stage, respectively. In this way, Equation (1) for j−1 allows us to obtain the following expression for the suction pressure of the *j* compression stage, $P_{2j-1}=P_{2\left(j-1\right)+1}$, in terms of the pressure drop coefficient of the j−1 intercooler and the outlet pressure of the j−1 compression stage,
  (2) $$P_{2j-1}=\left(1-\varepsilon_{j-1}\right)P_{2\left(j-1\right)},\;\;\mathrm{for}\;\;j=1,\ldots ,N_{c}$$
  The above equation is valid if we define $\varepsilon_{0}=0$ and therefore $P_{0}=P_{1}$.
- The gas temperature at the inlet of each compressor is not assumed to be the same. However, the compressed gas outlet temperature of each intercooler is close to $T_{1}$.
  (3) $$T_{2j+1}\neq T_{1},\;\;\mathrm{for}\;\;j=1,\ldots ,N_{c}-1$$
- The isentropic efficiencies of the individual compressors are assumed to be different, and the compression from 2j−1 to 2j is considered to occur at constant isentropic efficiency,
  (4) $$\eta_{SIC,j}=\frac{w_{j,s}}{w_{j}},\;\;\mathrm{for}\;\;j=1,...,N_{c}-1$$
  where $w_{j,s}$ and $w_{j}$ are the isentropic and actual adiabatic specific works provided to the *j* compression stage, respectively. The ideal compression work conducted on the *j* compression process corresponds to the work conducted on an isentropic compression process beginning at the same initial state and proceeding to the same final pressure (but not the same final state) as the actual compression process.

## 3. Theoretical Model

### 3.1. Optimal Interstage Pressures for Minimum Compression Specific Work

The aim of this section is to determine an expression for the optimal interstage pressures, which minimizes the specific compression work, in terms of the overall compression pressure ratio, considering pressures losses in the intercoolers, different outlet intercooling temperatures, and different isentropic efficiencies in each compression stage.

The total work provided to the $N_{c}$-multistage compressor is equal to the sum of the work supplied to each *j*-th compression stage

(5) $$w_{c}=\sum_{j=1}^{j=N_{c}}\frac{c_{P}T_{2j-1}}{\eta_{\mathrm{SIC},j}}\left[{\left(\frac{P_{2j}}{P_{2j-1}}\right)}^{x}-1\right]$$

where x=1−1/γ. The substitution of Equation (2) into Equation (5) derives an expression for the *j* compression work in terms of the discharge pressures of *j*-th compression stage and the j−1 intercooler pressure drop coefficient.

The total compression work is a multi-variable function of the interstage pressures, $w_{c}:R^{N_{c}-1}\to R$. Since $P_{2j}$ appears only at the numerator of the *j*-th terms and at the denominator of the $\left(j+1\right)$ term, the partial derivatives of $w_{c}$ with respect to each interstage pressure are given by

(6) $$\frac{\partial w_{c}}{x\partial P_{2j}}=\frac{xT_{2j-1}}{\eta_{SIC,j}P_{2j}}{\left[\frac{P_{2j}}{\left(1-\varepsilon_{j-1}\right)P_{2\left(j-1\right)}}\right]}^{x}-\frac{xT_{2j+1}\left(1-\varepsilon_{j}\right)}{\eta_{SIC,j+1}P_{2\left(j+1\right)}}{\left[\frac{P_{2\left(j+1\right)}}{\left(1-\varepsilon_{j}\right)P_{2j}}\right]}^{x+1},\;\mathrm{for}\;j=1,\ldots ,N_{c}-1$$

The interstage pressures at which the partial derivatives of $w_{c}$ are equal to zero, when Equation (6) vanishes, are the optimal interstage pressures that minimize the compression work. Appendix A shows the algebraic steps to obtain the optimal interstage pressures in terms of their predecessor and successor pressures from $\partial w_{c}/\partial P_{2j}=0$, as established in the following equation

(7) $$P_{2j}^{2}={\left(\frac{\alpha_{j+1}}{\alpha_{j}}\right)}^{1/x}\left(\frac{1-\varepsilon_{j-1}}{1-\varepsilon_{j}}\right)P_{2\left(j-1\right)}P_{2\left(j+1\right)}\Leftrightarrow \frac{\alpha_{j}^{1/x}P_{2j}}{\left(1-\varepsilon_{j-1}\right)P_{2\left(j-1\right)}}=\frac{\alpha_{j+1}^{1/x}P_{2\left(j+1\right)}}{\left(1-\varepsilon_{j}\right)P_{2j}},\;\mathrm{for}\;j=1,\ldots ,N_{c}-1$$

where $\alpha_{j}=T_{2j-1}/\eta_{SIC,j}$. This equation is analogous to that obtained by Vadasz and Weiner [6] (Equation (31)), assuming the intercooler temperatures and the interstage isoentropic efficiencies are the same for each compression stage and considering the existence of pressure drops in the intercoolers.

The purpose of the paper is to express the interstage pressures minimizing the compression work in terms of the initial and final pressure values. From Equation (7), it is inferred that the total input work required by the $N_{c}-$multistage compressor is minimized when the $N_{c}-1$ interstage pressures are chosen so that the ratio $\frac{\alpha_{j}^{1/x}P_{2j}}{\left(1-\varepsilon_{j-1}\right)P_{2\left(j-1\right)}}$ remains constant from one compression stage to the next. Denoting this constant by *K*, we can write

(8) $$K=\frac{\alpha_{N_{c}}^{1/x}P_{2N_{c}}}{\left(1-\varepsilon_{N_{c}-1}\right)P_{2\left(N_{c}-1\right)}}=\ldots =\frac{\alpha_{j}^{1/x}P_{2j}}{\left(1-\varepsilon_{j-1}\right)P_{2\left(j-1\right)}}=\ldots =\frac{\alpha_{1}^{1/x}P_{2}}{\left(1-\varepsilon_{0}\right)P_{0}}$$

The product of all $N_{c}$ pressure ratios affected by $\alpha_{j}^{1/x}/\left(1-\varepsilon_{j-1}\right)$ cancels out the $N_{c}-1$ interstage pressures and leads to determining the value of *K*,

(9) $$K^{x}=T_{1}\frac{\tau_{N_{c},g}}{\epsilon_{N_{c},g}^{x}}\pi^{\frac{x}{N_{c}}}$$

where $\pi =P_{2N_{c}}/P_{0}$ is the overall compression pressure ratio, and $\tau_{N_{c},g}$ and $\epsilon_{N_{c},g}$ are, respectively, the geometric means for the sets $\left\{\alpha_{i}/T_{1}=T_{2i-1}/\left(T_{1}\eta_{SIC,i}\right),\;\;i=1,\ldots ,j\right\}$ and $\left\{1-\varepsilon_{i-1},\;i=1,\ldots ,N_{c}\right\}$. The expression of constant *K*—see Equation (8)—allows us to obtain an equation for the individual pressure ratios, $\pi_{j}=P_{2j}/P_{2j-1}=\alpha_{j}^{-1/x}K,\;\;\mathrm{for}\;j=1,\ldots ,N_{c}$. The combination of this last relation with Equation (7) leads to the following expression for the optimal interstage pressures:

(10) $$P_{2j}={\left[{\left(\frac{\theta_{N_{c},g}}{\theta_{j,g}}\frac{\vartheta_{j,g}}{\vartheta_{N_{c},g}}\right)}^{\frac{1}{x}}\frac{\epsilon_{j,g}}{\epsilon_{N_{c},g}}\right]}^{j}{\left(P_{0}^{N_{c}-j}P_{2N_{c}}^{j}\right)}^{\frac{1}{N_{c}}},\;\;\mathrm{for}\;j=1,...,N_{c}-1$$

where $\theta_{j,g}$ and $\vartheta_{j,g}$ for $j=1,\ldots ,N_{c}$ are the geometric means of the elements of the sets $\left\{T_{2i-1}/T_{1},\;i=1,\ldots ,j\right\}$ and $\left\{\eta_{SIC,i},\;i=1,\ldots ,j\right\}$, respectively. It can observed when the outlet temperature for all the intercooling processes is $T_{1}$ that there are no pressures losses in the $N_{c}-1$ intercoolers, and all the compression stages have the same isentropic efficiency; thus, $\theta_{j,g}=\vartheta_{j,g}=\varepsilon_{j,g}=1$ for $j=1,\ldots ,N_{c}$, and Equation (A8) is therefore reduced to the well-known expression for the interstage pressures

(11) $$P_{2j}={\left(P_{0}^{N_{c}-j}P_{2N_{c}}^{j}\right)}^{\frac{1}{N_{c}}},\;\;\mathrm{for}\;j=1,\ldots ,N_{c}-1$$

Appendix B presents an alternative way to compute the optimal interstage pressures for the minimum compression work (Equations (10) and (A17) are equivalent equations) by using successive substitutions to solve the system of recursive nonlinear equations given by Equation (7). In Appendix D, the mathematical induction proof technique is used to prove that the expression for the optimal interstage pressures, given by Equation (A17), holds for every natural number $N_{c}$.

### 3.2. Minimum Compression Specific Work

The minimum compression specific work is determined by substituting Equation (9) into Equation (5),

(12) $$w_{c,\min}=c_{p}N_{c}\tau_{N_{c},a}T_{1}\left(\frac{\tau_{N_{c},g}}{\tau_{N_{c},a}\epsilon_{N_{c},g}^{x}}\pi^{\frac{x}{N_{c}}}-1\right)$$

where $\tau_{N_{c},a}$ is the arithmetic mean of the elements of the set $\left\{\alpha_{i}/T_{1},\;i=1,\ldots ,N_{c}\right\}$. Since minimum work occurs for an isothermal compression, $\tau_{N_{c},a}$ and $\tau_{N_{c},g}$ indicate how close or far the $N_{c}$ compression process with intercooling is from this ideal process. For the same suction temperature and interstage isentropic efficiency in each compression stage, Equation (12) is analogous to that obtained by Vadasz and Weiner [6] (see Equation (31)) and Hernández et al. [12] (see Equation (11)), with and without intercooler pressure losses, respectively. Equation (12) also corresponds to the expression for the minimum compression work obtained recently by López-Paniagua et al. [13] (see Equation (34)) for the case in which each compression stage has a different isentropic compression efficiency and the same suction temperature and the intercoolers do not present pressure drops.

## 4. Applications

### 4.1. Estimation of the Number of Compression Stages

The combination of Equations (8) and (9) leads to $\alpha_{j}^{1/x}\pi_{j}=K=(T_{1}\tau_{N_{c},g}/\epsilon_{N_{c}},$$g^{x}{)}^{1/x}\pi^{1/N_{c}}$. This relation implies that the individual and overall compression pressure ratios are proportional. The constant of proportionality is a geometric mean of the product of terms involving the pressure drops in the intercoolers, and the deviations of the suction temperatures and isentropic efficiencies of the compression stages from the suction temperature and isentropic efficiency of the *j*-th compression stage, respectively.

(13) $$\xi_{j}^{\frac{1}{xN_{c}}}=\pi_{j}/\pi^{1/N_{c}},\;\mathrm{where}\;\xi_{j}=\prod_{i=1}^{i=N_{c}}\left(T_{2i-1}/T_{2j-1}\right){\left(\eta_{SIC,i}/\eta_{SIC,j}\right)}^{-1}{\left(1-\varepsilon_{i-1}\right)}^{-x}$$

$\xi_{j}^{1/N_{c}}$ is the geometric mean of the elements of the set $\left\{\left(T_{2i-1}/T_{2j-1}\right){\left(\eta_{SIC,i}/\eta_{SIC,j}\right)}^{-1}{\left(1-\varepsilon_{i-1}\right)}^{-x}:i=1,\ldots ,N_{c}\right\}$, and it can be understood as a loss coefficient. When $\xi_{j}=1$, it is indicated that there are not pressure drops in the intercoolers and that the suction temperatures and isentropic efficiencies are all equal to the values corresponding to the *j*-th compression stage. Once a multistage compressor with intercooling is operating and taking the fits compressor as reference (j=1), $T_{2i-1}/T_{1}>1$, ${\left(1-\varepsilon_{i}\right)}^{-x}>1$, and $\eta_{SIC,i}/\eta_{SIC,1}\approx 1$; therefore, Equation (13) indicates that $\xi_{1}^{1/x}>1$.

From Equation (13), an expression is obtained to estimate the number of compression stages in terms of the *j*-th individual and overall compression pressure ratios and the coefficient $\xi_{j}^{1/x}$, as shown in the following equation

(14) $$N_{c}=\frac{\ln\pi +\ln\xi_{j}^{\frac{1}{x}}}{\ln\pi_{j}}$$

For all the compression stages, the individual and overall compression pressure ratios satisfy that $1\leq \pi_{j}\leq \pi$. According to this inequality and Equation (13), the loss coefficient can only take values defined over a bounded interval, $\pi^{-1}\leq \xi_{j}^{1/x}\leq \pi^{N_{c}-1}$. In this way, it should be noted that even if Equation (14) is strictly a nonlinear equation because $\xi_{j}^{1/x}$ is function of $N_{c}$, this equation together with the inequality for $\xi_{j}^{1/x}$ allows one to establish the upper and lower limits for the number of compression stages in the presence of pressure drops in the intercoolers and deviations of the suction temperatures and isentropic efficiencies from those of the *j*-th compression stage,

(15) $$2\frac{\ln\pi }{\ln\pi_{j}\pi }\leq N_{c}\leq 2\frac{\ln\pi }{\ln\pi_{j}}$$

**Figure 3:**
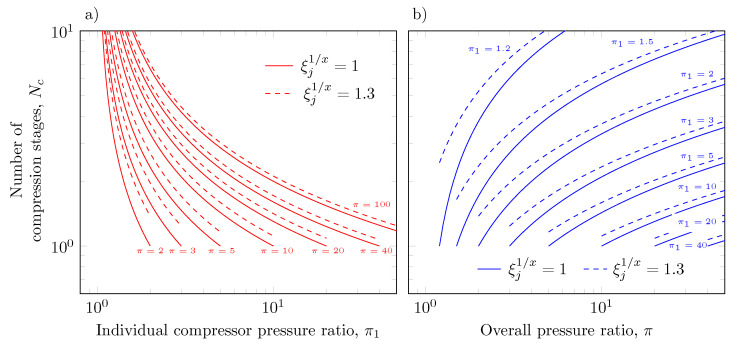
Number of compression stages and number of intercooling stages as a function: (**a**) individual compressor pressure ratio and (**b**) overall pressure ratio.

The determination of the number of compression stages is relevant for sizing a multistage compressor with intercooling during the design process. Figure 3 presents the estimation of the number of compression stages from Equation (14) by assuming the selected compressors are all of the same model (same individual pressure ratio, $\pi_{1}=\pi_{j}$), and for the cases in which the suction temperature deviations with respect to $T_{1}$ pressure drops in the intercoolers, and different isentropic efficiencies are taken ($\xi_{1}^{1/x}=1.3$) and not taken ($\xi_{1}^{1/x}=1$) into account.

Figure 3a,b show that for low overall pressure ratios and for a given individual pressure ratio, the number of compression stages exhibits the greatest dependence on the coefficient $\xi_{j}^{1/x}$. Despite this observation, this figure suggests that to reach the required discharge pressure, the number of compression stages must be selected assuming $\xi_{j}^{1/x}>1$.

### 4.2. Interstage Pressure Estimation of a Natural Gas Compression System

**Figure 4:**
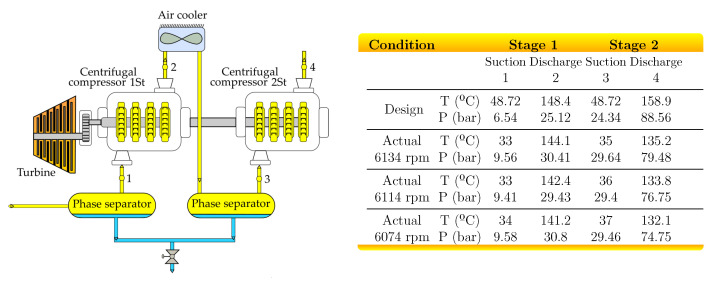
Natural gas two-stage centrifugal compressor: site design, and actual operating conditions.

Figure 4 presents a natural gas compression system composed of two Nouvo Pignone BCL608 centrifugal compressors, a soloair intercooler, and a scrubber to separate the condensates after intercooling. The two-stage compressor has a flow processing capacity of 132 MMSCFD of natural gas. The compressors are mounted on the same shaft and are driven mechanically by a low-pressure gas turbine, PGT25, which is driven by an aeroderivative gas turbine, GE 7LM-2500PE. Even when the mass flow through both compressors is not equal due to the phase separator, molecular masses and heat capacities of the natural gas are different at each state, and the working fluid in real compressors is far away from the ideal one, Equation (10) is applied to estimate the interstage pressure of the two-stages compressor system with intercooling. The motivation of this case study is to present such analytical expression as a short, simple, practical, and useful tool to obtain a first approximation of the interstage pressures of multistage compressors with intercooling, especially when the information required to obtain a rigorous estimation is not available.

To evaluate the pertinence of the use of Equation (10), the computed results were obtained using the following properties of natural gas entering the compression system: molecular weight of 26.54 kg/kmol, heat capacity of 1.446 kJ/kg·K, and molar fraction as presented in Table 1.

The computed results from Equation (10) were compared with those presented in Table A1, which were obtained from simulations of the centrifugal compression system in Aspen-Hysys, using the Peng-–Robinson equation of state as a thermodynamic model, the natural composition in Table 1, and the operating conditions presented in Figure 4.

The interstage pressure is computed with the following four equations, which are derived from Equation (10) by considering that in all cases, the isentropic efficiency of both compressors is the same:

- $P_{2}=\sqrt{P_{1}P_{4}}$, same suction temperatures ($T_{3}\approx T_{1}$) and no pressure drops in the intercooler ($\varepsilon_{1}\approx 0$);
- $P_{2}=\sqrt{\frac{P_{1}P_{4}}{1-\varepsilon_{1}}}$, same suction temperatures ($T_{3}\approx T_{1}$) and pressure drops in the intercooler;
- $P_{2}=\sqrt{{\left(\frac{T_{3}}{T_{1}}\right)}^{\frac{1}{x}}P_{1}P_{4}}$, different suction temperatures ($T_{3}\neq T_{1}$) and no pressure drops in the intercooler ($\varepsilon_{1}\approx 0$);
- $P_{2}=\sqrt{{\left(\frac{T_{3}}{T_{1}}\right)}^{\frac{1}{x}}\frac{P_{1}P_{4}}{1-\varepsilon_{1}}}$, different suction temperatures ($T_{3}\neq T_{1}$) and pressure drops in the intercooler;
- $P_{2}=\sqrt{{\left(\frac{T_{3}}{T_{1}}\cdot \frac{\eta_{SIC,1}}{\eta_{SIC,2}}\right)}^{\frac{1}{x}}\frac{P_{1}P_{4}}{1-\varepsilon_{1}}}$, different suction temperatures ($T_{3}\neq T_{1}$) and pressure drops in the intercooler.

**Figure 5:**
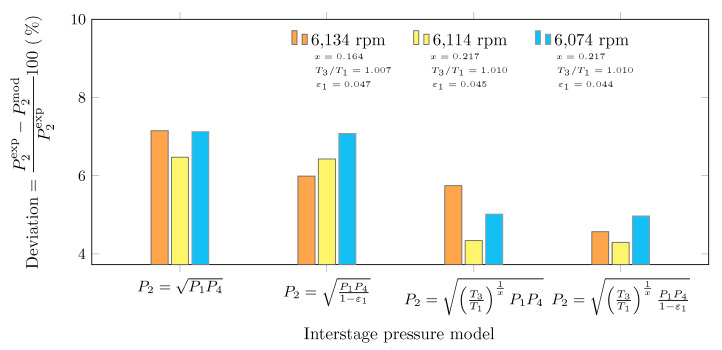
Percentage deviations in interstage pressure models under design and actual operating conditions.

Figure 5 shows the percentage deviations of the estimated interstage pressures with respect to the simulated ones at different operating conditions. For the four operating conditions, equation $P_{2}=\sqrt{{\left(T_{3}/T_{1}\right)}^{1/x}P_{1}P_{4}/\left(1-\varepsilon_{1}\right)}$ provides the lowest deviations, 4.57, 4.30, and 4.97% for 6134, 6114, and 6074 rpm under actual conditions, respectively. In contrast, the highest deviations correspond to the estimation of the interstage pressure by the geometric mean of the suction and discharge pressure of the complete compression system, $P_{2}=\sqrt{P_{1}P_{4}}$: 7.15, 6.47, and 7.13% for 6134, 6114, and 6074 rpm under actual conditions, respectively.

## 5. Conclusions

A general analytical expression for the interstage pressures minimizing the work supplied to a multistage compression system with intercooling is deduced, assuming different isentropic efficiencies and different suction gas temperatures in all the compression stages and the existence of pressure drops in the intercoolers. The optimal interstage pressures correspond to the geometric mean of the suction and discharge pressures corrected by terms involving the geometric means of the pressure drops in the intercoolers, the deviation of suction inter-temperatures from $T_{1}$, and the isentropic efficiencies of each compression stage. The application of the optimal interstage pressure expression indicates that the different isentropic efficiencies and suction gas temperatures in all the compression stages as well as the existence of pressure drops in the intercoolers could be relevant for the estimation of the number of compression stages for low overall pressure ratios. Finally, the use of the obtained expression to compute the interstage pressures of a two-stage centrifugal compressor of natural gas provides a suitable first approximation, especially when measurements of intermediate pressures are not available.

## Figures and Tables

**Table 1:** Natural gas molar fraction.

Component	CH_4_	C_2_H_6_	C_3_H_8_	iC_4_H_10_	nC_4_H_10_	iC_5_H_12_	N_2_	O_2_	H_2_O	CO_2_	H_2_S
$x_{i}$	0.3038	0.0594	0.0328	0.0043	0.0126	0.0036	0.543	0.0019	0.007	0.015	0.0044

## Nomenclature

$c_{P}$	specific heat at constant pressure, kJ kg${}^{-1}$ K${}^{-1}$
$c_{v}$	specific heat at constant volume, kJ kg${}^{-1}$ K${}^{-1}$
*K*	real constant defined by $K={\left(\frac{T_{2j-1}}{\eta_{SIC,j}}\right)}^{\frac{1}{x}}\frac{P_{2j}}{\left(1-\varepsilon_{j-1}\right)P_{2\left(j-1\right)}}$ for $j=1,\ldots ,N_{c}$
$N_{c}$	compression stages
*P*	pressures, bar
*R*	specific gas constant, kJ kg${}^{-1}$ K${}^{-1}$
*s*	specific entropy, kJ kg${}^{-1}$ K${}^{-1}$
*T*	temperature, K or ${}^{\circ }$C
*w*	specific work, kJ kg${}^{-1}$
*x*	$x=R/c_{P}=\left(\gamma -1\right)/\gamma$
*Greek symbols*	
$\alpha_{j}$	$\alpha_{j}^{x}=\frac{T_{2j-1}}{\eta_{SIC,j}}$, K${}^{\frac{1}{x}}$
Δ	drop or increment
$\varepsilon_{j}$	pressure drop coefficient across the *j*-intercooler, $\varepsilon_{j}=\frac{P_{2j}-P_{2j+1}}{P_{2j}}$
$\epsilon_{g}$	geometric mean for the set $\left\{1-\varepsilon_{j-1},\;\;j=1,\ldots ,N_{c}\right\}$
γ	adiabatic index or specific heat ratio
η	efficiency
π	pressure ratio
$\tau_{a}$	arithmetic mean for the set $\left\{T_{2j-1}/\eta_{SIC,j},\;\;j=1,\ldots ,N_{c}\right\}$, K
$\tau_{g}$	geometric mean for the set $\left\{T_{2j-1}/\eta_{SIC,j},\;\;j=1,\ldots ,N_{c}\right\}$, K
*Subscripts*	
*c*	compressor
*d*	discharge state
i,j,k,m	thermodynamic states
*s*	suction state
SIC	isentropic compression

## Appendix A. Optimal Interstage Pressures in Terms of Their Predecessor and Successor Pressures

The minimization problem for the compression work is formulated using $\left\{P_{2j}:j=1,\ldots ,N_{c}-1\right\}$ as independent variables and the partial derivatives of the compression work with respect to the independent variables given by Equation (6). The first step to determine interstage pressures minimizing the compression work in terms of the initial and final pressure values is to solve $\partial w_{c}/\partial P_{2j}=0$, implying that

(A1) $$P_{2j}^{2x}=\frac{T_{2j+1}\eta_{j}}{T_{2j-1}\eta_{j+1}}{\left(\frac{1-\varepsilon_{j-1}}{1-\varepsilon_{j}}P_{2\left(j-1\right)}P_{2\left(j+1\right)}\right)}^{x},\;\;\mathrm{for}\;\;j=1,\ldots ,N_{c}-1$$

Making j←j−1 and j←j+1 in Equation (A1),

(A2a) $$\begin{array}{cc} P_{2\left(j-1\right)}^{2x} & =\frac{T_{2j-1}\eta_{j-1}}{T_{2j-3}\eta_{j}}{\left(\frac{1-\varepsilon_{j-2}}{1-\varepsilon_{j-1}}P_{2\left(j-2\right)}P_{2j}\right)}^{x},\;\;\mathrm{for}\;\;j=1,\ldots ,N_{c} \end{array}$$

(A2b) $$\begin{array}{cc} P_{2\left(j+1\right)}^{2x} & =\frac{T_{2j+3}\eta_{j+1}}{T_{2j+1}\eta_{j+2}}{\left(\frac{1-\varepsilon_{j}}{1-\varepsilon_{j+1}}P_{2j}P_{2\left(j+2\right)}\right)}^{x},\;\;\mathrm{for}\;\;j=1,\ldots ,N_{c} \end{array}$$

The following expression is obtained by substituting Equations (A2a) and (A2b) into Equation (A1) and by using Equation (2):

(A3) $$P_{2j}^{2x}=\frac{T_{2j+1}\eta_{j}}{T_{2j-1}\eta_{j+1}}{\left(\frac{1-\varepsilon_{j-1}}{1-\varepsilon_{j}}P_{2\left(j-1\right)}P_{2\left(j+1\right)}\right)}^{x},\;\;\mathrm{for}\;\;j=1,...,N_{c}-1$$

Equation (7) is obtained from this last equation, and the definition of $\alpha_{j}=T_{2j-1}/\eta_{SIC,j}$.

## Appendix B. Optimal Interstage Pressures Obtained by Successive Substitutions

Equation (7) is a recurrence relation for the interstage pressure $P_{2j}$ in terms of its predecessor and successor interstage pressures, $P_{2\left(j-1\right)}$ and $P_{2\left(j+1\right)}$, respectively. However, this equation has no practical use, since this work assumes that the only measured pressures of the $N_{c}$-multistage compression system with intercooling are the suction ($P_{0}=P_{1}$) and discharge ($P_{2N_{c}}$) pressures. Equation (7) conforms a system of recursive nonlinear equations for $j=1,\ldots ,N_{c}$. This system is solved by first substituting $P_{2j}^{2}$, given by Equation (7), into Equation (7) for j=j+1 in order to obtain a relation for $P_{2\left(j+1\right)}^{3}$ in terms of $P_{2\left(j-1\right)}$ and $P_{2\left(j+2\right)}^{2}$; this expression is then used in Equation (7) for j=j+2 to derive an expression of $P_{2\left(j+3\right)}^{4}$ as a function of $P_{2\left(j-1\right)}$ and $P_{\left(j+3\right)}^{3}$. These successive substitutions are continued until the following expression for $P_{2\left(j+k-1\right)}$ in terms of $P_{2\left(j-1\right)}$ and $P_{2\left(j+k\right)}$ is inferred:

(A4) $$\begin{array}{cc} P_{2\left(j+k-1\right)}^{k+1} & ={\left(\frac{\alpha_{j+k}^{1/x}}{1-\varepsilon_{j+k-1}}\right)}^{k}\prod_{i=1}^{i=k}\frac{1-\varepsilon_{j+\left(i-2\right)}}{\alpha_{j+\left(i-1\right)}^{1/x}}P_{2\left(j-1\right)}P_{2\left(j+k\right)}^{k},\;\;\mathrm{for}\;\;k=1,...,N_{c}-1\;\;\mathrm{and}\;\;j=1,\ldots ,N_{c}-1 \end{array}$$

In the induction proof of Equation (A4), the mathematical induction proof technique is used to prove Equation (A4) regarding the integer *k*. Making j=1 and $k=N_{c}-m$, Equation (A4) becomes

(A5) $$\begin{array}{cc} P_{2\left(N_{c}-m\right)}^{N_{c}-m+1} & ={\left(\frac{\alpha_{N_{c}-m+1}^{1/x}}{1-\varepsilon_{N_{c}-m}}\right)}^{N_{c}-m}\prod_{i=1}^{i=N_{c}-m}\frac{1-\varepsilon_{i-1}}{\alpha_{i}^{1/x}}P_{0}P_{2\left(N_{c}-m+1\right)}^{N_{c}-m},\;\;\mathrm{for}\;\;m=1,\ldots ,N_{c}-1 \end{array}$$

After multiplying Equation (A5) by $\prod_{i=1}^{i=m}\left(\frac{1-\varepsilon_{N_{c}-i}}{\alpha_{N_{c}-i+1}^{1/x}}\frac{\alpha_{N_{c}-i+1}^{1/x}}{1-\varepsilon_{N_{c}-i}}\right)=1$, we obtain the following expression:

(A6) $$\begin{array}{cc} P_{2\left(N_{c}-m\right)}^{N_{c}-m+1} & ={\left(\frac{\alpha_{N_{c}-m+1}^{1/x}}{1-\varepsilon_{N_{c}-m}}\right)}^{N_{c}-m}\prod_{i=1}^{i=m}\frac{\alpha_{N_{c}-i+1}^{1/x}}{1-\varepsilon_{N_{c}-i}}\prod_{i=1}^{i=N_{c}}\frac{1-\varepsilon_{i-1}}{\alpha_{i}^{1/x}}P_{0}P_{2\left(N_{c}-m+1\right)}^{N_{c}-m},\;\;\mathrm{for}\;\;m=1,\ldots ,N_{c}-1 \end{array}$$

From Equation (A6) and pursuing the inductive approach presented in Appendix C, the following expression for the interstage pressure $P_{2\left(N_{c}-m\right)}$ in terms of the suction and discharge pressures is obtained:

(A7) $$\begin{array}{c} P_{2\left(N_{c}-m\right)}^{N_{c}}={\left(\prod_{i=1}^{i=m}\frac{\alpha_{N_{c}-i+1}^{1/x}}{1-\varepsilon_{N_{c}-i}}\right)}^{N_{c}}{\left(\prod_{i=1}^{i=N_{c}}\frac{1-\varepsilon_{i-1}}{\alpha_{i}^{1/x}}\right)}^{m}P_{0}^{m}P_{2N_{c}}^{N_{c}-m},\;\;\mathrm{for}\;\;m=1,\ldots ,N_{c}-1 \end{array}$$

The expression for the interstage pressure $P_{2j}$ is computed by making $k=N_{c}-i+1$ in the first product of Equation (A7) and $j=N_{n}-m$ in the overall expression

(A8) $$\begin{array}{c} P_{2j}^{N_{c}}={\left(\prod_{i=1}^{i=k}\frac{1-\varepsilon_{i-1}}{\alpha_{i}^{1/x}}\right)}^{N_{c}}{\left(\prod_{i=1}^{i=N_{c}}\frac{\alpha_{i}^{1/x}}{1-\varepsilon_{i-1}}\right)}^{j}P_{0}^{N_{c}-j}P_{2N_{c}}^{j},\;\;\mathrm{for}\;\;j=1,\ldots ,N_{c}-1 \end{array}$$

From the definition of $\alpha_{j}^{1/x}$, Equation (A8) can be rewritten as

(A9) $$P_{2j}^{N_{c}}={\left[\prod_{k=1}^{N_{c}}\frac{T_{2k-1}}{\eta_{SIC,k}{\left(1-\varepsilon_{k-1}\right)}^{x}}\right]}^{\frac{j}{x}}{\left[\prod_{k=1}^{j}\frac{\eta_{SIC,k}{\left(1-\varepsilon_{k-1}\right)}^{x}}{T_{2k-1}}\right]}^{\frac{N_{c}}{x}}P_{0}^{N_{c}-j}P_{2N_{c}}^{j}$$

## Appendix C. Induction Proof of Equation (A4)

In this Appendix, we prove Equation (A4) by mathematical induction about the integer *k*. The essential steps of the proof are the proof for k=1 (base case) and the inductive step, in which the equation for k+1 is proved, assuming Equation (A4) is valid for *k* (induction hypothesis).

- Base case: When k=1, Equation (A4) corresponds to Equation (7), proving Equation (A4) is true for k=1.
- Induction hypothesis: In this step, we assume Equation (A4) is valid for *k*.
- Inductive step: When j=j+k, Equation (7) raised to the power of k+1 becomes
  (A10) $$P_{2\left(j+k\right)}^{2\left(k+1\right)}={\left(\frac{\alpha_{j+k+1}^{1/x}}{\alpha_{j+k}^{1/x}}\right)}^{k+1}{\left(\frac{1-\varepsilon_{j+k-1}}{1-\varepsilon_{j+k}}\right)}^{k+1}P_{2\left(j+k-1\right)}^{k+1}P_{2\left(j+k+1\right)}^{k+1}$$
  The substitution of Equation (A4), corresponding to the induction hypothesis, into the left-hand side of Equation (A10) leads to
  (A11) $$P_{2\left(j+k\right)}^{2\left(k+1\right)}={\left(\frac{\alpha_{j+k+1}^{1/x}}{\alpha_{j+k}^{1/x}}\right)}^{k+1}{\left(\frac{1-\varepsilon_{j+k-1}}{1-\varepsilon_{j+k}}\right)}^{k+1}{\left(\frac{\alpha_{j+k}^{1/x}}{1-\varepsilon_{j+k-1}}\right)}^{k}\prod_{i=1}^{i=k}\frac{1-\varepsilon_{j+\left(i-2\right)}}{\alpha_{j+\left(i-1\right)}^{1/x}}P_{2\left(j-1\right)}P_{2\left(j+k\right)}^{k}P_{2\left(j+k+1\right)}^{k+1}$$
  After performing some algebraic steps in the above equation, we obtain the following expression:
  (A12) $$P_{2\left(j+k\right)}^{k+2}={\left(\frac{\alpha_{j+k+1}^{1/x}}{1-\varepsilon_{j+k}}\right)}^{k+1}\prod_{i=1}^{i=k+1}\frac{1-\varepsilon_{j+\left(i-2\right)}}{\alpha_{j+\left(i-1\right)}^{1/x}}P_{2\left(j-1\right)}P_{2\left(j+k+1\right)}^{k+1}$$
  Thus, Equation (A4) holds for k+1, and the proof of induction step is complete.

## Appendix D. Induction proof of Equation (A7)

In this appendix, we prove by induction that for $m=1,\ldots ,N_{c}-1$, Equation (A7) is true.

- Base case: When j=1 and $k=N_{c}-1$, Equation (A4) corresponds to Equation (A7), proving Equation (A7) holds for m=1.
- Induction hypothesis: In this step, we assume Equation (A7) is valid for *m*.
- Inductive step: For m=m+1, Equation (A6) becomes
  (A13) $$\begin{array}{cc} P_{2\left(N_{c}-m-1\right)}^{N_{c}-m}\; & ={\left(\frac{\alpha_{N_{c}-m}^{1/x}}{1-\varepsilon_{N_{c}-m-1}}\right)}^{N_{c}-m-1}\prod_{i=1}^{i=m+1}\frac{\alpha_{N_{c}-i+1}^{1/x}}{1-\varepsilon_{N_{c}-i}}\prod_{i=1}^{i=N_{c}}\frac{1-\varepsilon_{i-1}}{\alpha_{i}^{1/x}}P_{0}P_{2\left(N_{c}-m\right)}^{N_{c}-m-1} \end{array}$$
  Raising Equation (A7), corresponding to the induction hypothesis, to the power of $\left(N_{c}-m-1\right)/N_{c}$, leads to
  (A14) $$\begin{array}{c} P_{2\left(N_{c}-m\right)}^{N_{c}-m-1}={\left(\prod_{i=1}^{i=m}\frac{\alpha_{N_{c}-i+1}^{1/x}}{1-\varepsilon_{N_{c}-i}}\right)}^{N_{c}-m-1}{\left(\prod_{i=1}^{i=N_{c}}\frac{1-\varepsilon_{i-1}}{\alpha_{i}^{1/x}}\right)}^{\frac{m\left(N_{c}-m-1\right)}{N_{c}}}P_{0}^{\frac{m\left(N_{c}-m-1\right)}{N_{c}}}P_{2N_{c}}^{\left(N_{c}-m\right)\frac{N_{c}-m-1}{N_{c}}} \end{array}$$
  Substituting Equation (A7) into the left-hand side of Equation (A15)
  (A15) $$\begin{array}{c} P_{2\left(N_{c}-m-1\right)}^{N_{c}-m}=\prod_{i=1}^{i=m+1}\frac{\alpha_{N_{c}-i+1}^{1/x}}{1-\varepsilon_{N_{c}-i}}{\left(\frac{\alpha_{N_{c}-m}^{1/x}}{1-\varepsilon_{N_{c}-m-1}}\prod_{i=1}^{i=m}\frac{\alpha_{N_{c}-i+1}^{1/x}}{1-\varepsilon_{N_{c}-i}}\right)}^{N_{c}-m-1}\\ {\left(\prod_{i=1}^{i=N_{c}}\frac{1-\varepsilon_{i-1}}{\alpha_{i}^{1/x}}\right)}^{\frac{m\left(N_{c}-m-1\right)}{N_{c}}+1}P_{0}^{\frac{m\left(N_{c}-m-1\right)}{N_{c}}+1}P_{2N_{c}}^{\frac{\left(N_{c}-m\right)\left(N_{c}-m-1\right)}{N_{c}}} \end{array}$$
  After carrying out some algebra with the above equation, we derive the following expression:
  (A16) $$P_{2\left(N_{c}-m-1\right)}^{N_{c}-m}={\left(\prod_{i=1}^{i=m+1}\frac{\alpha_{N_{c}-i+1}^{1/x}}{1-\varepsilon_{N_{c}-i}}\right)}^{N_{c}-m}{\left[{\left(\prod_{i=1}^{i=N_{c}}\frac{1-\varepsilon_{i-1}}{\alpha_{i}^{1/x}}\right)}^{m+1}P_{0}^{m+1}P_{2N_{c}}^{N_{c}-\left(m+1\right)}\right]}^{\frac{N_{c}-m}{N_{c}}}$$
  Raising Equation (A16) to the power of $N_{c}/\left(N_{c}-m\right)$, we may write
  (A17) $$P_{2\left[N_{c}-\left(m+1\right)\right]}^{N_{c}}={\left(\prod_{i=1}^{i=m+1}\frac{\alpha_{N_{c}-i+1}^{1/x}}{1-\varepsilon_{N_{c}-i}}\right)}^{N_{c}}{\left(\prod_{i=1}^{i=N_{c}}\frac{1-\varepsilon_{i-1}}{\alpha_{i}^{1/x}}\right)}^{m+1}P_{0}^{m+1}P_{2N_{c}}^{N_{c}-\left(m+1\right)}$$
  Thus, Equation (A7) holds for m+1, and the proof of induction step is complete.

## Appendix E. Two-Stage Compression System With Intercooling

Table A1 presents some results of the ASPEN-HYSIS simulation for the two-stage centrifugal compressor using the Peng–Robinson equation of state as thermodynamic model for the natural gas, whose molar composition is presented in Table 1.

**Table A1:** Thermodynamic states of the natural gas two-stage compression system obtained from ASPEN-HYSIS simulations for different shaft speeds.

		6134 rpm						6114 rpm						6074 rpm					
	$\dot{m}$ $\left(\frac{\mathrm{kg}}{h}\right)$	*T* (°C)	*P* (bar)	*ρ* $\left(\frac{\mathrm{kg}}{m^{3}}\right)$	*c_p_* $\left(\frac{\mathrm{kJ}}{\mathrm{kg}K}\right)$	*MW* $\left(\frac{\mathrm{kg}}{\mathrm{kmol}}\right)$	*Z* (-)	*T* (°C)	*P* (bar)	*ρ* $\left(\frac{\mathrm{kg}}{m^{3}}\right)$	*c_p_* $\left(\frac{\mathrm{kJ}}{\mathrm{kg}K}\right)$	*MW* $\left(\frac{\mathrm{kg}}{\mathrm{kmol}}\right)$	*Z* (-)	*T* (°C)	*P* (bar)	*ρ* $\left(\frac{\mathrm{kg}}{m^{3}}\right)$	*c_p_* $\left(\frac{\mathrm{kJ}}{\mathrm{kg}K}\right)$	*MW* $\left(\frac{\mathrm{kg}}{\mathrm{kmol}}\right)$	*Z* (-)
1	5.52	33	10.58	10.16	1.45	26.54	0.98	33	10.43	9.99	1.45	26.54	0.98	34	10.59	10.14	1.45	26.54	0.98
2	5.52	144.1	31.42	23.51	1.64	26.54	0.99	142.4	30.44	22.85	1.63	26.54	0.99	142.2	30.50	22.9	1.63	26.54	0.99
2’	5.52	35	30.65	32.38	1.53	26.54	0.95	36	30.41	31.98	1.53	26.54	0.95	37	30.47	31.92	1.53	26.54	0.95
3	5.50	35	30.65	32.28	1.52	26.58	0.95	36	30.41	31.89	1.52	26.58	0.95	37	30.47	31.83	1.52	26.58	0.95
4	5.50	135.2	80.49	63.53	1.71	26.58	0.98	133.8	77.76	61.58	1.70	26.58	0.98	132.1	75.76	60.26	1.70	26.58	0.98
